# A Sequenced Study of Improved Dielectric Properties of Carbon Nanotubes and Metal Oxide-Reinforced Polymer Composites

**DOI:** 10.3390/ma15134592

**Published:** 2022-06-29

**Authors:** Faiza, Memoona Qammar, Safi Ullah Butt, Zahida Malik, Ahmad Aziz Alahamadi, Abraiz Khattak

**Affiliations:** 1 Department of Chemistry, National University of Sciences and Technology (NUST), H-12, Islamabad 44100, Pakistan; faizaijaz710@gmail.com (F.); mqammar@connect.ust.hk (M.Q.); 2 Department of Chemistry, COMSATS University Islamabad, Lahore Campus Defence Road off Riwind Road, Lahore 51500, Pakistan; 3 High Voltage Laboratory, United States-Pakistan Center for Advanced Studies in Energy, National University of Sciences and Technology (NUST), Sector H-12, Islamabad 44000, Pakistan; safibutt541@gmail.com; 4 Department of Chemistry, The Hong Kong University of Science and Technology (HKUST), Clear Water Bay Rd., Kowloon 999077, Hong Kong; 5 School of Chemistry, Faculty of Engineering & Physical Sciences, University of Southampton, Highfield Campus, Southamoton SO17 1BJ, UK; 6 Department of Electrical Engineering, College of Engineering, Taif University KSA, P.O. Box 11099, Taif 21944, Saudi Arabia; aziz@tu.edu.sa

**Keywords:** properties, mechanical properties, bandgap tuning, carbon nanotubes, copper oxide, nickel oxide

## Abstract

Polymers have gained attraction at the industrial level owing to their elastic and lightweight nature, as well as their astonishing mechanical and electrical applications. Their scope is limited due to their organic nature, which eventually leads to the degradation of their properties. The aim of this work was to produce polymer composites with finely dispersed metal oxide nanofillers and carbon nanotubes (CNTs) for the investigation of their charge-storage applications. This work reports the preparation of different polymeric composites with varying concentrations of metal oxide (MO) nanofillers and single-walled carbon nanotubes (SWCNTs). The successful synthesis of nanofillers (i.e., NiO and CuO) was carried out via the sonication and precipitation methods, respectively. After, the smooth and uniform polymeric composite thin films were prepared via the solution-casting methodology. Spectroscopy and diffraction techniques were used for the preliminary characterization. Scanning electron microscopy was used to check the dispersion of carbon nanotubes (CNTs) and MOs in the polymer matrix. The addition of nanofillers and carbon nanotubes (CNTs) tuned the bandgap, reduced the strain, and enhanced the elastic limit of the polymer. The addition of CNT enhanced the mechanical strength of the composite; however, it increased the conductivity, which was tuned by using metal oxides. By increasing the concentration of NiO and CuO from 2% to 6% bandgap of PVA, which is 5–6 eV reduced to 4.41 and 4.34 eV, Young’s moduli of up to 59 and 57.7 MPa, respectively, were achieved. Moreover, improved dielectric properties were achieved, which shows that the addition of metal oxide enhances the dielectric behavior of the material.

## 1. Introduction

Polymeric materials are being extensively used at the industrial level by virtue of their elastic nature, light weight, and tremendous mechanical, electrical, and dielectric properties, even at elevated temperatures [1]. The organic nature of polymers causes a degradation in their properties due to environmental factors, which limits their applications [2]. This problem has created the need for materials that have greater stability against environmental stresses. To resolve this issue, the fabrication of polymeric nanocomposites is under consideration, and especially the usage of inorganic nanofillers, which not only provide greater stability against the environment, but also enhance their electrical and dielectric properties [3,4,5]. Different morphologies of nanofiller are being explored to achieve a greater interface area. Various methods are being employed to prepare polymer nanocomposites (e.g., solution casting, injection molding, compression molding, melt blending, etc.) [1,6,7,8]. The surface functionalization of fillers enhances the extent of the dispersion in the polymer matrix. To achieve the best results from nanofillers, the key factor is to achieve a good dispersion of the nanofiller in the polymeric matrix. Several analysis techniques are available to probe and investigate these materials; for example, Fourier transform infrared (FTIR) spectroscopy, thermal gravimetric analysis (TGA), differential scanning calorimetry (DSC), and other spectroscopic techniques [9,10,11,12].

In previous studies, many inorganic fillers, such as metals and metal oxides, were employed to enhance different physical properties of the polymers (for example, the addition of Cu(Cu_2_O) to the polyethylene enhanced the electrical conductivity about five times [13]). Similarly, in other studies, the incorporation of filler electrical, thermal, and mechanical properties of epoxy significantly improved [14]. The polymers/MOs are showing their remarkable performances in the field of optoelectronics [15]. Likewise, several studies that have been previously conducted prove the effect of inorganic fillers on different polymers, such as Polydimethylsiloxane (PDMS), Poly(vinylidene fluoride) (PVDF), and Poly(vinyl chloride) (PVC) [16,17,18,19]. In previous research, the polyolefins were reinforced with BaTiO_3_, TiO_2_, and ZrO_2_ and exhibited high charge-storing abilities [19]. Many composites are also being used to inhibit corrosive behavior [20]. When metal oxides are added to the polymer matrix, they reduce the Young’s modulus of the polymer [21]. To balance this, carbon nanotubes can be added, which helps to enhance the mechanical properties, as well as escalate the electrical and thermal stabilities [22].

E. Flahaut et al. reported a composite of carbon nanotubes and metal oxides. They deployed hot pressing to fabricate the composites by using metals such as Fe, Co, or Fe/Co, and oxides such as Al_2_O_3_, MgO, and Al_2_O_4_. Techniques such as X-ray diffraction (XRD) and scanning electron microscopy (SEM) were used for their characterization and comparison with the nanocomposite having zero carbon content. The transverse fracture strength, fracture toughness, and relative densities were estimated by the three-point bending test and Archimedes method. The authors observed electrical conductivities of the composites in the range from 0.2 to 0.4 Scm^−1^, and a reduction in the CNT content in the composite by the hot-press method [23]. In 2002, A. Peigney et al. prepared ceramic and carbon nanotube composites, including CNT–Co–MgO, CNT–Fe–Al_2_O_3_ and CNT–Fe/Co–MgAl_2_O_3_, by using the high-temperature-extrusion method. Mono-phased oxide solution was prepared by chemical routes, followed by attrition via ball milling and the reduction in metal oxides under H_2_–CH_4_, and the maximum temperature was 1050 °C. A dense composite was formed by high-temperature extrusion in a graphite die, where Al_2_O_3_ and MgAl_2_O_4_ were extruded at 1500 °C, but, for MgO, the temperature was surged to 1730 °C [24].

Generally, composites exhibit high permittivity and energy density if the filler has high permittivity compared with the polymer matrix. If the dielectric contrast in the permittivity is large between the two phases, then this leads to poor dielectric strength; thus, many researchers are working to improve the interfacial modulus of the filler and polymer matrix [25]. Recently, Giustino and Pasquarell developed a theory that explains the variation in the dielectric permittivity with respect to the interfacial boundaries [26]. PVA has a carbon backbone and significant water solubility, which makes it suitable for many applications, such as drug delivery, catalysis, medicine, etc. [27].

The goals of the conducted work were to prepare a polymer nanocomposite with a good dispersion of nanofiller and CNTs in the polymer matrix, and to investigate their charge-storing abilities so that the material may be considered for high-power-density capacitors. The prepared polymer composite would be the amalgam of the advantages of both the polymer matrix, being lightweight, easy to process, and low cost, and metal oxide, having permittivity, but being brittle in nature. The SWCNTs enhance the mechanical strength (e.g., elasticity), Young’s modulus, and stiffness of the composite. In this way, the combination of all the fillers and matrix will overcome the gap found in the literature.

## 2. Experimental

### 2.1. Materials and Chemicals

Carboxyl-modified single-walled carbon nanotubes (SWCNTs) were purchased from He, Ji Inc, Tainan, Taiwan. The solvents, such as sulfuric acid, H_2_SO_4_ (97% pure), hydrochloric acid (HCl), and nitric acid (HNO_3_) (67% pure), of analytical grade, were purchased from Sigma Aldrich (St. Louis, MI, USA). Nickle nitrate hexahydrate (NiNO_3_·6H_2_O) and copper nitrate tri hydrate (Cu (NO_3_)_2_·3H_2_O) of RDH grade were also provided by Sigma Aldrich. Powdered polyvinyl alcohol (PVA) of commercial grade was used. All chemicals except SWCNTs were used as received, without further purification.

### 2.2. Preparation of CNT/PVA Composites

#### 2.2.1. Purification and Oxidation of SWCNTs

SWCNTs were purified to eliminate any amorphous carbon and metal residues by using the already reported method [28]. Well-ground SWCNTs were added to a 3 M HCl solution and were heated at 70 °C on a hot plate. After that, the mixture was filtered with a 0.22 µm PTFE filter, washed with ultrapure water, and, eventually, the nanotubes were vacuum-dried at ambient temperature to obtain pure powder.

Before making the polymers, the SWCNTs needed to be functionalized to achieve dispersion in the polymer matrix, and so the abovementioned pure and dried powder was added to the solvent mixture with a 3:1 ratio of H_2_SO_4_ and HNO_3_, and the whole mixture was heated under the same conditions mentioned above. After 4 h, the mixture was cooled and was washed with ultrapure water. The purified and oxidized SWCNTs were filtered, washed, and vacuum-dried for 48 h at ambient temperature.

#### 2.2.2. Preparation of Metal Oxides (MOs)

Nickle oxide (NiO) nanoparticles were prepared by modifying the sonication method [29], where a 2 M NaOH(aq) solution was added dropwise to the 1 M aqueous solution of NiCl_2_·6H_2_O under constant stirring. The entire solution was sonicated in a probe sonicator for 60 min. The product was subsequently filtered, washed, and centrifuged. The product was dried at 250 °C to eliminate any residue left. Finally, the product was calcined at a 500 °C temperature for 3 h to obtain small crystallite and particle size [30].

Copper oxide (CuO) nanoparticles were synthesized by the slight modification of the already reported precipitation method [31]. In short, an aqueous solution with 0.02 M Cu (NO_3_)_2_·3H_2_O and 1 mL glacial acid was refluxed and heated at 100 °C. NaOH was rapidly added to the abovementioned solution to adjust the pH from 6–7, and, suddenly, large numbers of black precipitates were formed. They were filtered, washed, and air-dried under ambient conditions.

#### 2.2.3. Composite Formation (PVA/MO/SWCNTs)

Aqueous solutions (1 mL) of well-ground and purified SWCNTs and MOs were sonicated overnight in a sonication bath of 100 W, having a frequency of 40 kHz to ensure a homogeneous suspension formation. The 5% aqueous solution of PVA was prepared, and the SWCNT/MO mixture was added. In our composites, the concentration of the PVA solution and quantity of SWCNTs was kept constant, but the concentration of MOs varied to make different composites. The details on all the samples are summarized in Table 1.

#### 2.2.4. Solution-Casting Method

Thin films of all samples were fabricated by using the typical solution-casting method, in which the abovementioned suspensions were stirred for 7 h to obtain homogeneity. The resulting homogenous and viscous solution was poured onto the glass substrate, followed by vacuum-drying at 55 °C for 17 h.

### 2.3. Characterization

**Figure 1:**
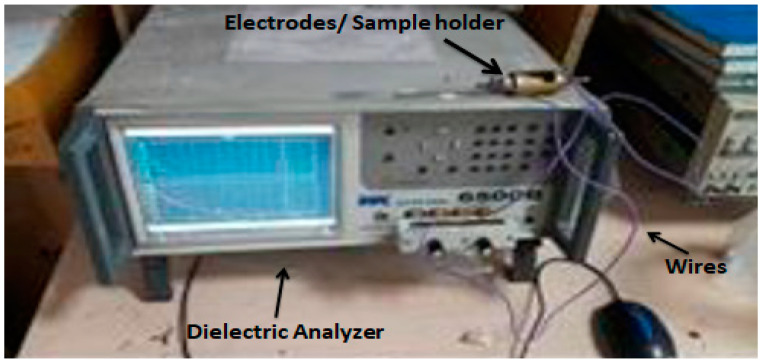
Picture of Dielectric Analyzer (WK 6500) Measurement Setup.

X-ray powder diffraction (XRPD) was performed by employing a model STOE Germany X-ray diffractometer operating at 40 kV, with Cu Kα radiation, and within a 10° ≤ 2θ ≥ 80° range for powder and film samples. Field emission scanning electron microscopy (FESEM) analysis was performed on MIRA3 TESCAN Zeiss supra 55 VP for qualitative and morphological testing of powder and film samples after Au coating. Atomic force microscopy (AFM) was deployed for topographical analysis of samples, and 3-D images were captured by using a high-resolution high-performance environmental scanning probe microscope (JSPM-5200), WINSPM version 5, in the tapping mode. Fourier transform infrared spectroscopy (FTIR) was performed by using the platinum ATR model alpha, with a spectral range of 4000–550 cm^−1^, and by placing the samples directly onto the diamond scanner. UV–VIS (single beam) spectrophotometer model T60 PG instruments, UK, was employed to check the transmittance and confirm the bandgaps through the absorbance coefficient. For the dielectric properties, the Wayne Kerr model 6500 B, with a frequency range from 100 Hz to 5 MHz, was used. Thin films were cut into circles of 2 mm diameters and pressed between two electrodes of an LCR meter to record the spectra. For the measurement of the dielectric properties, RC parallel configuration, with a bias voltage of 5 Vrms, was used, as shown in Figure 1. The measurements were taken at room temperature and pressure. The circular samples, of 0.1 mm thicknesses and 13 mm diameters (according to the holder dimension), were cut from the prepared discs. Tensile testing was performed using the standard ASTM D638-12 of UTM (AG-X Plus), Shimadzu, Japan, for the sample, with a gauge length of 20 mm and a width of 1 mm. A force of 10 N was applied, and the speed was kept constant at 5 mm/min.

## 3. Results and Discussion

### 3.1. Conformational Analysis

**Figure 2:**
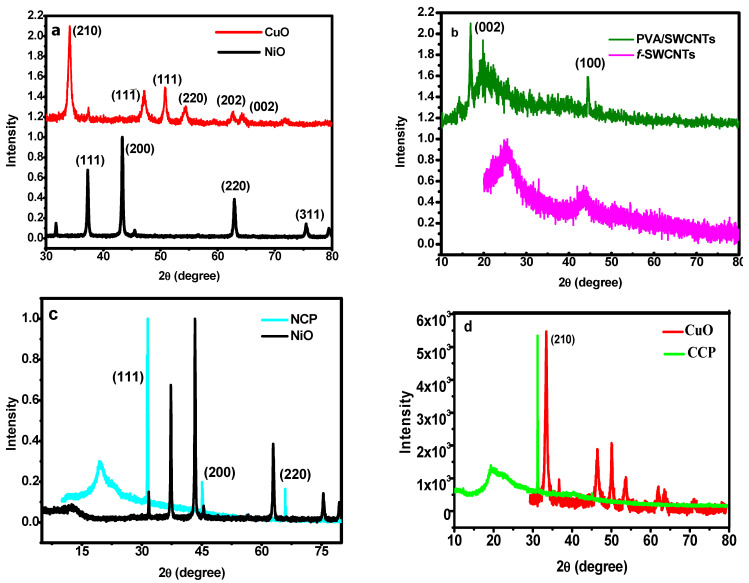
XRD patterns of: (**a**) CuO and NiO; (**b**) pure *f*-SWCNTs and PVA/SWCNTs; (**c**) NiO and NCP3; (**d**) CuO and CCP3.

The crystallinity, phase purity, and successful synthesis of NiO and CuO were investigated by XRD. Figure 2a shows the XRD patterns for NiO and CuO.

In the case of NiO, the diffraction peaks are observed at 20°, 37.2°, 43.2°, and 63.0°, and they can be related to the (111), (200), and (220) planes, respectively, and this can be indexed to the JCPDS 78-0429 [32]. These results clearly indicate the cubic structure and *Fm*$\bar{3}$*m* space group. Similarly, in the case of CuO, no additional impurity peak can be observed. The XRD pattern can be well indexed to the monoclinic structure and the JCPDS 80-1268 [33]. The peak broadening can be attributed to the small size of the particles. The XRD pattern of the pure functionalized SWCNTs and the composite of the SWCNTs/PVA were compared to investigate whether any structural change occurred, as shown in Figure 2b. The structure of the SWCNTs remained intact, and, in the XRD of the composite, the peaks of all the constituents can be observed. Finally, the XRD analysis of the MO/SWCNT/PVA composites was also carried out to ensure the structural persistence of each component in the composite. The XRD patterns of NiO/SWCNTs/PVA (NCP) and CuO/SWCNTs/PVA (CCP), in comparison with pure NiO and CuO, are shown in Figure 2c,d, respectively. It can be clearly observed that the MO crystals perfectly aligned towards one plane, which is evidence of a fine dispersion and homogenous orientation. The peaks for all the components, including PVA, can be observed [34].

**Figure 3:**
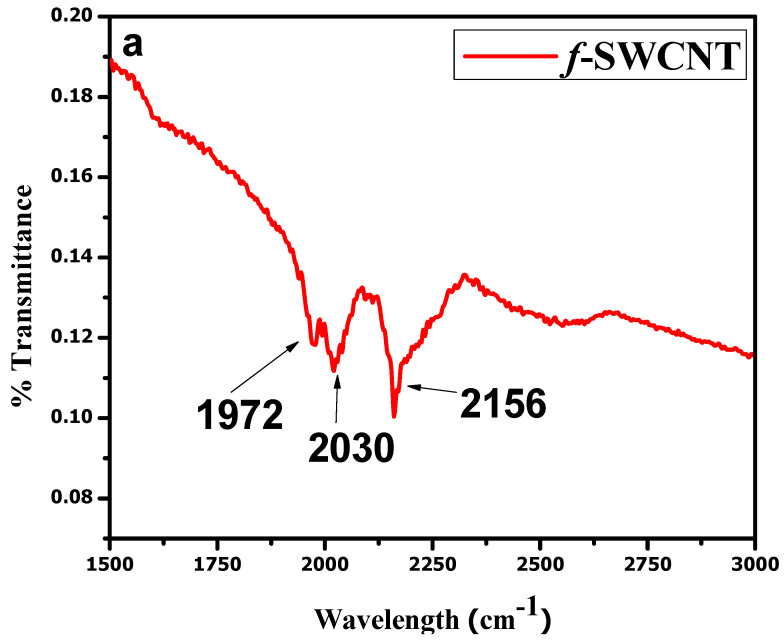

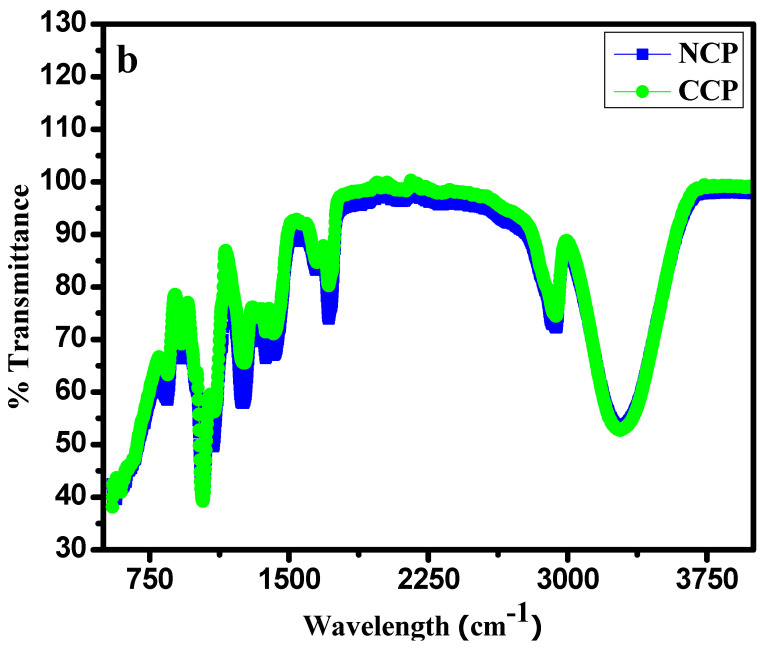
FTIR analyses of: (**a**) *f*-SWCNTs; (**b**) NCP and CCP.

The FTIR analysis presented in Figure 3a is evidence of the successful purification and functionalization of the SWCNTs. An intense band of nitrile (−C≡N) was observed near 2030 cm^−1^, and a C=O band at 1972 cm^−1^. Various bands of nitrates were observed between 1738 and 2136 cm^−1^. A band of an aliphatic C–H stretch was also observed near 2156 cm^−1^. The data show the successful functionalization of the SWCNTs. FTIR analyses for the NCP and CCP composites were also carried out and are shown in Figure 3b [35]. All the characteristic bands of each constituent can be seen in the IR spectra of the composites [32,33,36].

**Figure 4:**
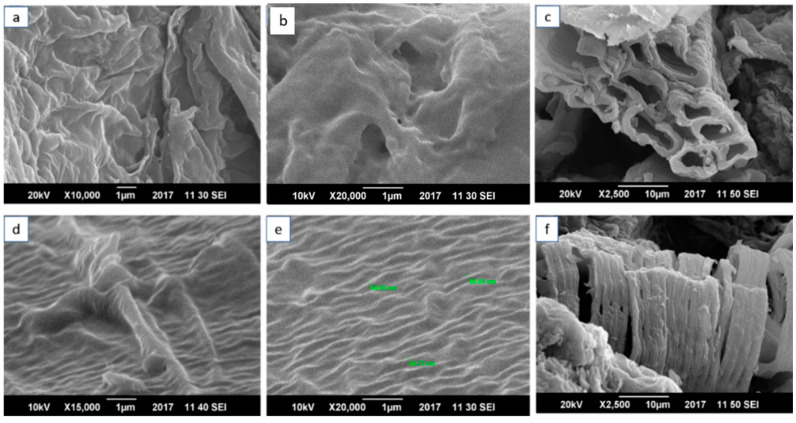
Scanning-electron-microscopy images of (**a**–**c**) NCP at 1µm resolution for (**a**,**b**) at voltages of 20 kv and at 10 µm for (**c**) at 10 kv; (**d**–**f**) CCP at 1µm resolution for (**d**,**e**) at voltages of 20 kv and at 10 µm for (**f**) at 10 kv.

The dispersion of MOs and alignment of the SWCNTs was analyzed by SEM. Figure 4 shows the SEM micrographs of the NCP and CCP, analyzed at different resolutions. Figure 4a,b presents the micrographs of the NCP at magnifications of X10,000 and X20,000, respectively. These images were taken at a specimen distance of 1 µm, and at voltages of 20 kV and 10 kV for a and b, respectively. It was necessary to proceed with the analysis at a lower voltage to avoid the film rupturing. In Figure 4c, with a specimen distance of 10 µm, a clear image of the zigzag CNT is observed. Figure 4d–f shows the composite CCP with a homogeneous dispersion of *f*-SWCNTs. For Figure 4e, the diameter was calculated, which ranged from 44 to 88 nm for the zigzag SWCNTs dispersed in the PVA. The SEM micrographs show the zigzag morphology and alignment of the SWCNTs, which resulted in a very fine dispersion. The SWNTs have uniform dimensions and embedment throughout the polymer matrix, as shown in Figure 3.

**Figure 5:**
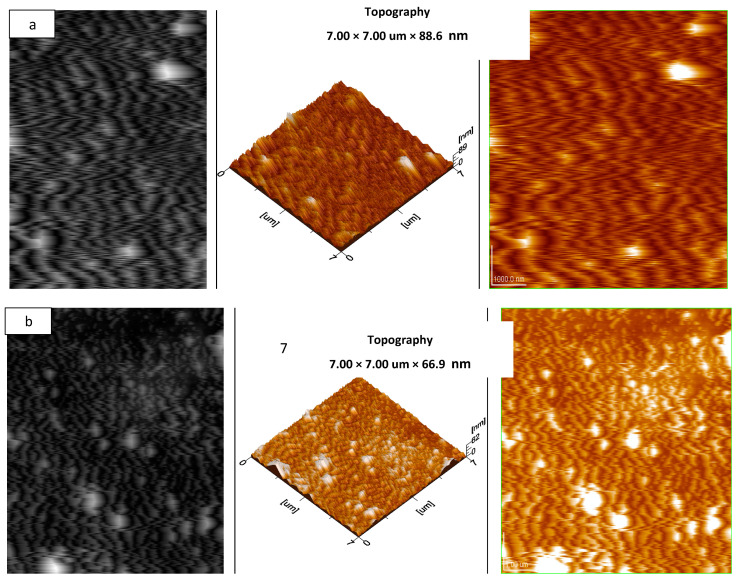
AFM images of: (**a**) NCP 3 and (**b**) CCP 3.

AFM was preformed to obtain 3-D images of the composites. The analysis was performed under taping mode, and the 3-D images show the topography of the *f*-SWCNTs. The 3-D images obtained by AFM also confirm the uniform dispersion of the zigzag SWCNTs and metal oxides in the PVA films. Figure 5 shows 3-D images of (a) NCP and (b) CCP.

### 3.2. Physical Properties

**Figure 6:**
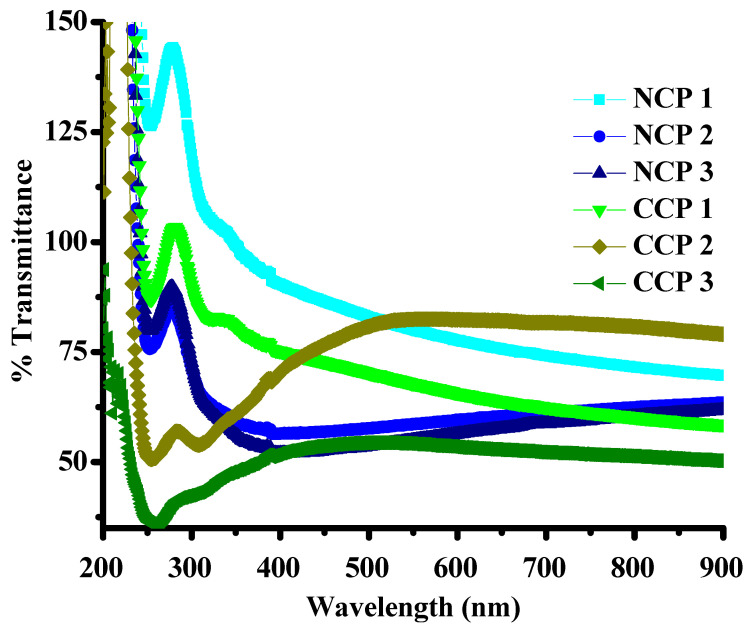
Transmittance of the composites.

The optical properties, including the transmission and molar absorption coefficients of the composites, were investigated by using UV–VIS spectrophotometry. The effects of the metal oxides and SWCNTs on the bandgap energies were thoroughly investigated by plotting Tauc’s plots for all the composites. It is clear from Figure 6 that the transmittance of the composites is inversely proportional to the concentration of SWCNTs and MOs.

**Figure 7:**
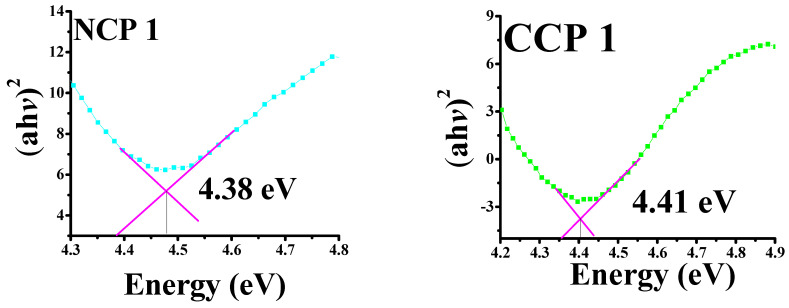

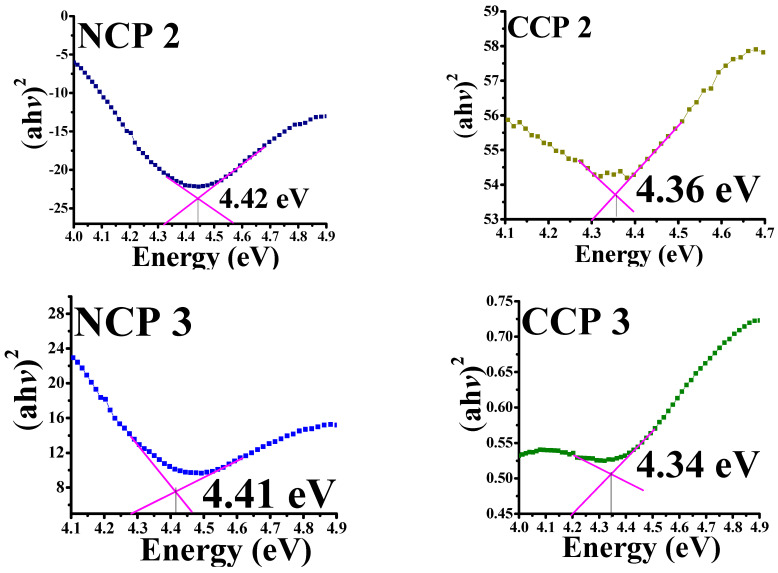
Tauc’s plots for bandgaps of all prepared samples.

The bandgap energies were calculated by plotting the absorbance coefficient ((ahv)^2^) versus the energy (hv), as shown in Figure 7.

It can be seen that, by incorporating NiO and CuO, the bandgap decreased for the PVA, which was 5–6 eV [37]. A decreasing trend in the bandgap energy was observed by an increase in the concentration of MOs, as the incorporation of CuO and NiO creates localized electronic states, which serve as recombination and trapping centers [38]. The bandgap variation along the material composition is summarized in Table 2.

**Figure 8:**
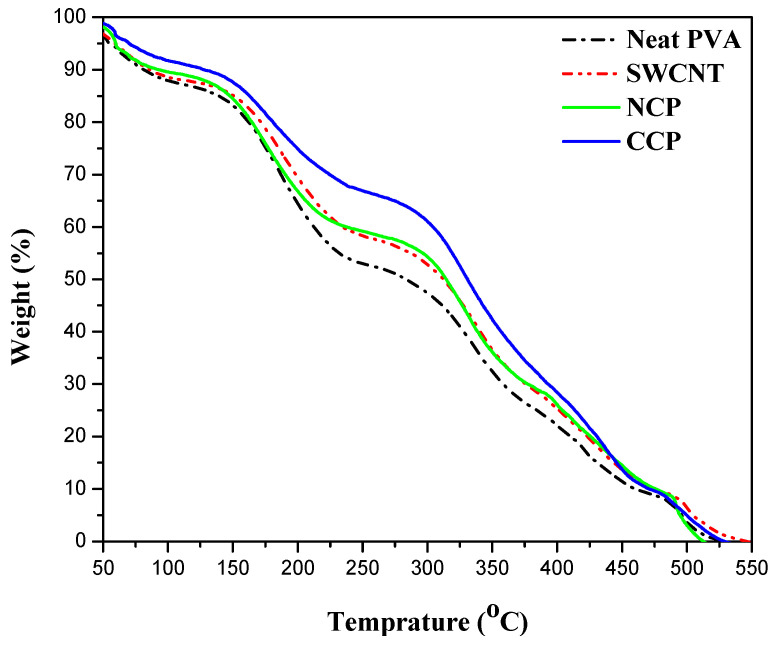
Comparison of thermal stabilities of neat PVA, SWCNTs, CCP 3, and NCP 3.

Thermogravimetric analysis was employed to assess the thermal stability of the CNPs and CCPs with the highest concentrations of nanofiller, and their comparison with neat-PVA and SWCNT thin films is given in Figure 8.

According to the decomposition pattern, three steps are involved in the complete decomposition of all the materials. In the case of neat PVA, almost 9% of the mass loss was observed during the first step up to 100 °C, which can be attributed to the evaporation of water and the removal of residual oxygen that might be trapped during processing [39]. In the second and third steps, the thermal decomposition of the polymer is involved. The degradation of the side chain takes place at 175 °C, and a 30% weight loss was observed. Further degradation of the polymer backbone occurs at 350 °C, with a 31% loss in weight. Finally, the oxidation step started at 500 °C, and 100% degradation takes place at 550 °C [40]. On the one hand, it can be seen that the extent of the degradation in the neat PVA is the maximum, and, in the case of the CNP, the degradation is the minimum, and it is also stable at a higher temperature compared with the neat, SWCNT, and CCP films. On the other hand, the CCP and SWCNT films showed almost comparable degradation. The best stability of the CNP is due to the higher thermal stability of NiO_2_ as compared with the CuO [41], which helps the material to retain its properties and structure against the temperature.

The mechanical properties were investigated by using a universal testing machine. Different parameters, such as the Young’s Modulus (E), elastic limit (S_y_), ultimate tensile strength (UTS), total strain (e_total_), elastic strain (e_elastic_), and toughness, were measured from the stress vs. strain graphs [35,37]. Typically, metal oxides have a brittle nature, but it can be tuned by the addition of PVA and SWCNTs because both possess a high elastic modulus and good elastic limit. The addition of metal oxides and SWCNTs into the polymer could be responsible for the reduced strain, enhanced toughness, and improved elasticity. All the parameters calculated from the stress vs. strain graph are summarized in Table 3. The variations in the behavior were due to the migration of metal oxides and SWCNTs, which require further optimization to achieve enhanced mechanical strength. However, a good enhancement in the mechanical parameters was still observed.

To study the practical application of the prepared samples, dielectric spectroscopy was carried out by investigating the complex permittivity in the frequency range from 100 Hz to 5 MHz. The log–log graphs were drawn for the sake of simplicity. Complex permittivity comprises the real part ($\varepsilon^{\prime }$) and imaginary part ($\varepsilon^{\prime \prime }$), and it is given in Equation (1):

(1) $$\;\varepsilon^{*}=\varepsilon^{\prime }-i\varepsilon^{\prime \prime }$$

The real part of complex permittivity defines the ability of a material to store energy, while the imaginary part defines the dielectric loss and represents the energy dissipation [42,43].

**Figure 9:**
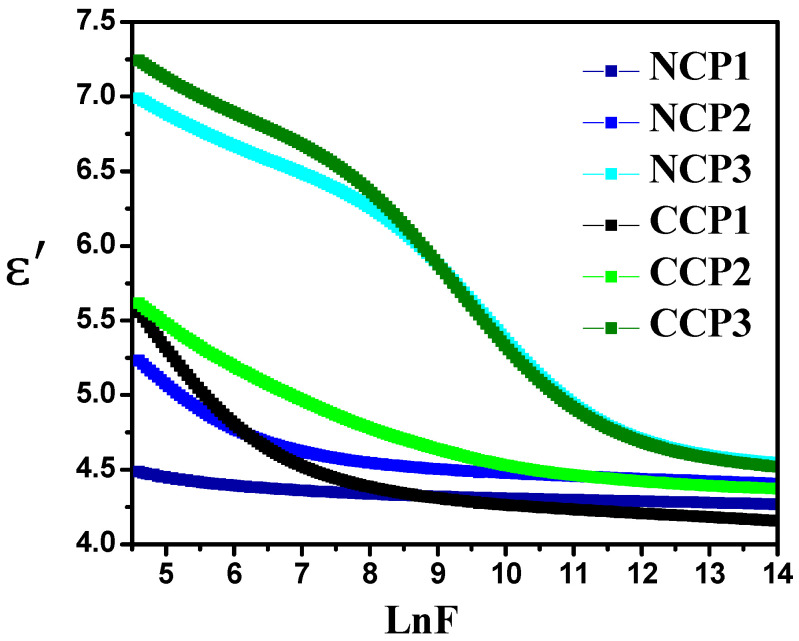
Variation in real part of permittivity with varying concentrations of nanofillers at different frequencies.

The real part of the permittivity can be calculated as:

(2) $$\varepsilon^{\prime }=\frac{C\times d}{A\;\times \varepsilon_{o}}\;$$

where *C* is the capacitance, *d* is the thickness (nm), *A* is the surface area (m^2^), and $\varepsilon_{o}\;\mathrm{is}\;\mathrm{the}\;permitivity\;of\;space\;\left(8.854\times 10-12\;F/m\right)$. A significant change in the value with the change in frequency can be seen in Figure 9. This change is due to the dipolar charge carriers among the two isolated states. It was observed that, with the increase in the frequency, the real part of the permitivity of the material decreased, as the dipoles were unable to align themselves due to the relaxation process, along with the field at a high frequency [44]. It is evident from the Figure 9 $\varepsilon^{\prime }$ that the addition of the filler improved the dielectric behavior of the prepared films, with CCP 3 expressing the highest value of the $\varepsilon^{\prime }$. The increase in the $\varepsilon^{\prime }$ value is due to the accumulation of mobile charge carriers at the interface of the filler polymer matrix.

The increase in the value of the permitivity with the increase in the concenteration of fillers is due to the interfacial polariazation of metal oxides and the polymer matrix. Dielectric loss is the imaginary part of the permittivity that shows the heat dissipation, and mathematically, it can be calculated as follows:

(3) $$\varepsilon^{\prime \prime }=\varepsilon^{\prime }\times Dissipation\;factor$$

**Figure 10:**
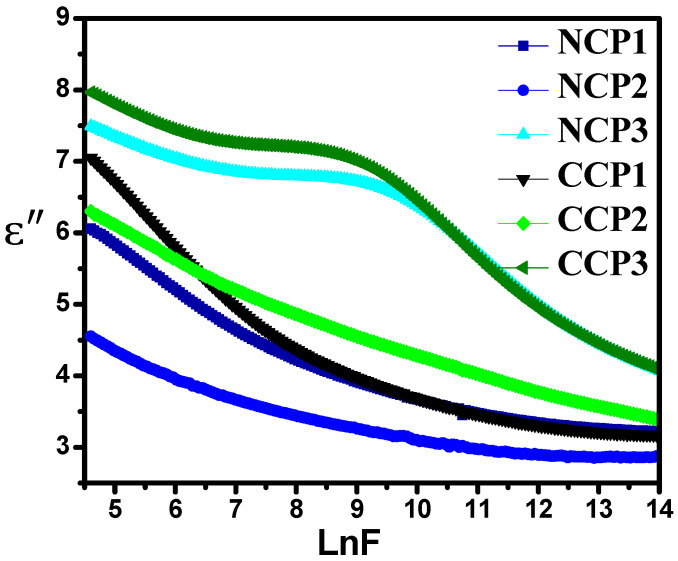
Variation in dielectric loss with varying concentrations of nanofillers at different frequencies.

It can be observed from Figure 10 that the value of the imaginary part of the permittivity$\;\left(\varepsilon^{\prime \prime }\right)$ decreases with the increase in the applied frequency. The heat losses may occur due to rapid α and β relaxation processes [45]. The motion of the charge carriers also causes dielectric heat loss.

This heat loss is actually the result of two pehenomena: the interfacial polarization of the charges between the electrodes, and their dipolar orientation. The motion of the charge carriers causes the decrease in both parts of the permittivities and results in an increase in the conductivity. The AC conductivity of the material is its ability to allow the passage of current. When the external field is applied on the material, they align themselves, and their hopping frequency resonates with the external frequency, which can be measured as in [46]:

(4) $$\sigma_{ac}=2\pi f\varepsilon^{\prime }\varepsilon_{o}tan\delta \;$$

**Figure 11:**
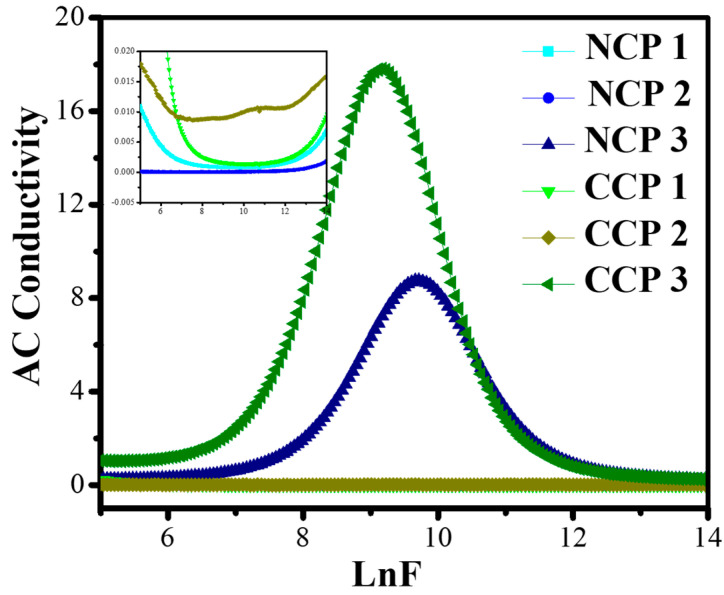
Variation in AC conductivity with varying concentrations of nanofillers at different frequencies.

It is evident from Figure 11 that the conductivity of all the samples increased with the increase in the frequency, and then decreased in the higher range.

The increase in the conductivity is due to the motion of the charge carriers through the composite. It can be seen from the graphs that CCP3 showed the best dielectric behavior among all the prepared composites.

## 4. Conclusions

The successful synthesis of nanofillers (i.e., NiO and CuO) was carried out via the sonication and precipitation methods, respectively, followed by the preparation of polymeric composite thin films via the solution-casting methodology. The dielectric, optical, thermal, and mechanical properties were investigated for the prepared nanocomposites. A comparison between the nanocomposites of NiO and CuO was also performed. It could be seen that the NiO nanocomposites (NCs) performed better than those of CuO. For the optical properties, the transmittance of the NiO nanocomposites was higher than that of the CuO nanocomposites. The NiO NCs expressed a bandgap value of 4.48; however, a reduction in the bandgap value was seen with the increase in the filler content. For the CuO NCs, it was observed that the bandgap was 4.41, which was reduced to 4.34 with the increase in the filler concentration. The Young’s modulus was calculated and it showed a decrease in the value with the increase in the concentration: CNP 3 and CCP 3 showed values of 59 and 57.7, respectively. The TGA expressed that the complete decomposition of the samples was achieved till 550 °C. The lowest temperature was seen for the neat PVA, and the highest thermal stability was observed for CNP3. The dielectric spectroscopy results revealed that the NiO NCs expressed higher dielectric constants than the CuO nanocomposites. For the NiO and CuO NCs, 6 wt.% filled samples expressed the highest dielectric constants, at 5 MHz with the NCP3 expressing, and the CCP3 expressing 6.68, respectively. Similar trends for the dielectric loss and AC conductivity were seen. It was concluded that the nanocomposites performed better than the neat PVA, and optimal results were seen for the samples with higher concentrations. By comparing the NiO and CuO nanocomposites, it was seen that the NiO NCs performed better than the CuO nanocomposites in all aspects. By changing the concentration of nanoparticles, different properties of polymer nanocomposites can be tuned according to the desired application in electrical or optical devices.

## Figures and Tables

**Table 1:** The concentrations of MOs in polymer/SWNT/MO composites.

Sr. no	Sample Code	Concentration of Reagents Used (wt.%)		
		SWCNTs	NiO	CuO
1	CCP 1	0.01	-	2
2	CCP 2	0.01	-	4
3	CCP 3	0.01	-	6
4	NCP 1	0.01	2	-
5	NCP 2	0.01	4	-
6	NCP 3	0.01	6	-

**Table 2:** The bandgap energies for the composites.

Sr. No.	Sample Code	Bandgap (eV)
1	CCP 1	4.41
2	CCP 2	4.36
3	CCP 3	4.34
4	NCP 1	4.48
5	NCP 2	4.42
6	NCP 3	4.41

**Table 3:** Summary of mechanical properties of composites.

Sr. No.	Sample	E (MPa)	S_y_ (MPa)	UTS (MPa)	e_total_	e_elastic_
1	CCP 1	62.5	3.2	20.5	1.12	1.02
2	CCP 2	22.4	1.2	11.3	1.17	1.10
3	CCP 3	57.7	4.3	13.02	0.51	0.41
4	CNP 1	28.3	1.62	7.2	0.50	0.44
5	CNP 2	31.3	3.9	14.9	0.74	0.63
6	CNP 3	59.0	5.44	10.04	0.49	0.45

## Data Availability

The data presented in this study are available upon request from the corresponding author.

## References

[1] Yan S., Verestek W., Zeizinger H., Schmauder S. (2021). Characterization of Cure Behavior in Epoxy Using Molecular Dynamics Simulation Compared with Dielectric Analysis and DSC. Polymers.

[2] Faiza F., Khattak A., Butt S., Imran K., Ulasyar A., Ali A., Khan Z., Mahmood A., Ullah N., Alahmadi A. (2021). Investigation of Hydrothermally Stressed Silicone Rubber/Silica Micro and Nanocomposite for the Coating High Voltage Insulation Applications. Materials.

[3] Khattak A., Amin M., Iqbal M., Abbas N. (2018). Life estimation and analysis of dielectric strength, hydrocarbon backbone and oxidation of high voltage multi stressed EPDM composites. Mater. Res. Express.

[4] Azevedo J.V., Dorp E.R.V., Hausnerova B., Möginger B. (2021). The Effects of Chain-Extending Cross-Linkers on the Mechanical and Thermal Properties of Poly(butylene adipate terephthalate)/Poly(lactic acid) Blown Films. Polymers.

[5] Elbayoumy E., El-Ghamaz N.A., Mohamed F.S., Diab M.A., Nakano T. (2021). Dielectric Permittivity, AC Electrical Conductivity and Conduction Mechanism of High Crosslinked-Vinyl Polymers and Their Pd(OAc)_2_ Composites. Polymers.

[6] Barber P., Balasubramanian S., Anguchamy Y., Gong S., Wibowo A., Gao H., Ploehn H.J., Zur Loye H.-C. (2009). Polymer Composite and Nanocomposite Dielectric Materials for Pulse Power Energy Storage. Materials.

[7] Işık B., Kurtoğlu A.E., Gürdağ G., Keçeli G. (2020). Radioactive cesium ion removal from wastewater using polymer metal oxide composites. J. Hazard. Mater..

[8] Arbatti M., Shan X., Cheng Z.-Y. (2007). Ceramic–Polymer Composites with High Dielectric Constant. Adv. Mater..

[9] Maharramov A.A., Ramazanov M.A., Di Palma L., Shirinova H.A., Hajiyeva F.V. (2018). Influence of Magnetite Nanoparticles on the Dielectric Properties of Metal Oxide/Polymer Nano-composites Based on Polypropylene. Russ. Phys. J..

[10] Yao X., Kou X., Qiu J., Moloney M.G. (2016). Generation Mechanism of Negative Dielectric Properties of Metallic Oxide Crystals/Polyaniline Composites. J. Phys. Chem. C.

[11] Kim P., Jones S.C., Hotchkiss P.J., Haddock J.N., Kippelen B., Marder S.R., Perry J.W. (2007). Phosphonic Acid-Modified Barium Titanate Polymer Nanocomposites with High Permittivity and Dielectric Strength. Adv. Mater..

[12] Demirezen S., Eroğlu A., Azizian-Kalandaragh Y., Altındal Ş. (2020). Electric and dielectric parameters in Au/n-Si (MS) capacitors with metal oxide-polymer interlayer as function of frequency and voltage. J. Mater. Sci. Mater. Electron..

[13] Ushakov N.M., Kosobudsky I.D. (2020). About the features of electric conductivity models for polymer composite nano-materials based on Cu (Cu_2_O)-LDPE. Semiconductors.

[14] Munkaila S., Bentley J., Schimmel K., Ahamad T., Alshehri S.M., Bastakoti B.P. (2021). Polymer directed synthesis of NiO nanoflowers to remove pollutant from wastewater. J. Mol. Liq..

[15] Ezzat H.A., Hegazy M.A., Nada N.A., Osman O., Ibrahim M.A. (2020). Development of natural polymer/metal oxide nanocomposite reinforced with graphene oxide for optoelectronic applications. NRIAG J. Astron. Geophys..

[16] Rehman M.N.U., Munawar T., Nadeem M.S., Mukhtar F., Maqbool A., Riaz M., Manzoor S., Ashiq M.N., Iqbal F. (2021). Facile synthesis and characterization of conducting polymer-metal oxide based core-shell PANI-Pr_2_O–NiO–Co_3_O_4_ nanocomposite: As electrode material for supercapacitor. Ceram. Int..

[17] Srivastava M., Surana K., Singh P.K., Singh R.C. (2021). Nickel Oxide embedded with Polymer Electrolyte as Efficient Hole Transport Material for Perovskite Solar Cell. Eng. Sci..

[18] Rani P., Ahamed B., Deshmukh K. (2021). Dielectric and electromagnetic interference shielding properties of zeolite 13X and carbon black nanoparticles based PVDF nanocomposites. J. Appl. Polym. Sci..

[19] Guo N., DiBenedetto S.A., Tewari P., Lanagan M.T., Ratner M.A., Marks T.J. (2010). Nanoparticle, size, shape, and interfacial effects on leakage current density, permittivity, and breakdown strength of metal oxide-polyolefin nanocomposites: Experiment and theory. Chem. Mater..

[20] Tishkevich D.I., Vorobjova A.I., Vinnik D.A. (2020). Formation and Corrosion Behavior of Nickel/Alumina Nanocomposites. Solid State Phenom..

[21] Yu L., Ranjan V., Nardelli M.B., Bernholc J. (2009). First-principles investigations of the dielectric properties of polypropylene/metal-oxide interfaces. Phys. Rev. B.

[22] Jordan J., Jacob K.I., Tannenbaum R., Sharaf M., Jasiuk I. (2005). Experimental trends in polymer nanocomposites—A review. Mater. Sci. Eng. A.

[23] Flahaut E., Peigney A., Laurent C., Marliere C., Chastel F., Rousset A. (2000). Carbon nanotube–metal–oxide nanocomposites: Microstructure, electrical conductivity and mechanical properties. Acta Mater..

[24] Peigney A., Flahaut E., Laurent C., Chastel F., Rousset A. (2002). Aligned carbon nanotubes in ceramic-matrix nanocomposites prepared by high-temperature extrusion. Chem. Phys. Lett..

[25] Li Z., Fredin L.A., Tewari P., DiBenedetto S.A., Lanagan M.T., Ratner M.A., Marks T.J. (2010). In Situ Catalytic Encapsulation of Core-Shell Nanoparticles Having Variable Shell Thickness: Dielectric and Energy Storage Properties of High-Permittivity Metal Oxide Nanocomposites. Chem. Mater..

[26] Giustino F., Pasquarello A. (2005). Theory of atomic-scale dielectric permittivity at insulator interfaces. Phys. Rev. B.

[27] Halima N.B. (2016). Poly (vinyl alcohol): Review of its promising applications and insights into biodegradation. RSC Adv..

[28] Ciobotaru C.C., Damian C.M., Iovu H. (2013). Single-wall carbon nanotubes purification and oxidation. UPB Sci. Bull. Ser. B Chem. Mater. Sci..

[29] Duraisamy N., Numan A., Fatin S.O., Ramesh K., Ramesh S. (2016). Facile sonochemical synthesis of nanostructured NiO with different particle sizes and its electrochemical properties for supercapacitor application. J. Colloid Interface Sci..

[30] Qammar M., Malik Z., Malik F., Baig T., Chaudhary A.J. (2019). Antibacterial activity of Mg1-xNixO (x= 0.5) nano-solid solution; experimental and computational approach. J. Mol. Struct..

[31] Zhu J., Li D., Chen H., Yang X., Lu L., Wang X. (2004). Highly dispersed CuO nanoparticles prepared by a novel quick-precipitation method. Mater. Lett..

[32] Davar F., Fereshteh Z., Salavati-Niasari M. (2009). Nanoparticles Ni and NiO: Synthesis, characterization and magnetic properties. J. Alloys Compd..

[33] Kouklin N., Tzolov M., Straus D., Yin A., Xu J.M. (2004). Infrared absorption properties of carbon nanotubes synthesized by chemical vapor deposition. Appl. Phys. Lett..

[34] Heitz T., Drevillon B., Godet C., Bouree J.E. (1998). Quantitative study of C—H bonding in polymerlike amorphous carbon films using in situ infrared ellipsometry. Phys. Rev. B.

[35] Barua S., Chattopadhyay P., Phukan M.M., Konwar B.K., Karak N. (2014). Hyperbranched epoxy/MWCNT-CuO-nystatin nanocomposite as a high performance, biocompatible, anti-microbial material. Mater. Res. Express.

[36] Liu D., Li J., Sun F., Xiao R., Guo Y., Song J. (2014). Liquid crystal microphase separation of cellulose nanocrystals in wet-spun PVA composite fibers. RSC Adv..

[37] Abdullah O., Aziz S.B., Omer K., Salih Y.M. (2015). Reducing the optical band gap of polyvinyl alcohol (PVA) based nanocomposite. J. Mater. Sci. Mater. Electron..

[38] Popovics S. (1973). A numerical approach to the complete stress-strain curve of concrete. Cem. Concr. Res..

[39] Hsu T.T., Slate F.O., Sturman G.M., Winter G. (1963). Microcracking of plain concrete and the shape of the stress-strain curve. ACI J. Proc..

[40] Gómez I., Otazo E., Hernández H., Rubio E., Varela J., Ramirez-Cardona M., Barajas I., Gordillo A. (2015). Thermal degradation study of PVA derivative with pendant phenylthionecarbamate groups by DSC/TGA and GC/MS. Polym. Degrad. Stab..

[41] Wang Y., Zhong M., Chen F., Yang J. (2009). Visible light photocatalytic activity of TiO_2_/D-PVA for MO degradation. Appl. Catal. B Environ..

[42] Mustafa E., Afia R.S.A., Nouini O., Tamus Z. (2021). Implementation of Non-Destructive Electrical Condition Monitoring Techniques on Low-Voltage Nuclear Cables: I. Irradiation Aging of EPR/CSPE Cables. Energies.

[43] Mustafa E., Afia R.S.A., Tamus Z.A. (2020). Application of Non-Destructive Condition Monitoring Techniques on Irradiated Low Voltage Unshielded Nuclear Power Cables. IEEE Access.

[44] El-Mallah H.M. (2012). AC electrical conductivity and dielectric properties of perovskite (Pb, Ca) TiO_3_ ceramic. Acta Phys. Pol.-Ser. A Gen. Phys..

[45] Roy A.S., Gupta S., Sindhu S., Parveen A. (2013). Ramamurthy, Dielectric properties of novel PVA/ZnO hybrid nanocomposite films. Compos. Part B Eng..

[46] Radoń A., Włodarczyk P., Drygała A., Łukowiec D. (2018). Electrical properties of epoxy nanocomposites containing Fe_3_O_4_ nanoparticles and Fe_3_O_4_ nanoparticles deposited on the surface of electrochemically exfoliated and oxidized graphite. Appl. Surf. Sci..

